# Macrophage-mediated inflammatory response decreases mycobacterial survival in mouse MSCs by augmenting NO production

**DOI:** 10.1038/srep27326

**Published:** 2016-06-02

**Authors:** Kun Yang, Yongjian Wu, Heping Xie, Miao Li, Siqi Ming, Liyan Li, Meiyu Li, Minhao Wu, Sitang Gong, Xi Huang

**Affiliations:** 1 Program of Immunology, Affiliated Guangzhou Women and Children’s Medical Center, Zhongshan School of Medicine, Sun Yat-sen University, Guangzhou 510080, China; 2 Institute of Tuberculosis Control, Key laboratory of Tropical Diseases Control (Sun Yat-sen University), Ministry of Education, Guangzhou 510080, China; 3 Department of Traditional Chinese Medicine, Third Affiliated Hospital, Sun Yat-sen University, Guangzhou 510630, China

## Abstract

*Mycobacterium tuberculosis* (MTB) is a hard-to-eradicate intracellular microbe, which escapes host immune attack during latent infection. Recent studies reveal that mesenchymal stem cells (MSCs) provide a protective niche for MTB to maintain latency. However, the regulation of mycobacterial residency in MSCs in the infectious microenvironment remains largely unknown. Here, we found that macrophage-mediated inflammatory response during MTB infection facilitated the clearance of bacilli residing in mouse MSCs. Higher inducible nitric oxide synthase (iNOS) expression and nitric oxide (NO) production were observed in mouse MSCs under macrophage-mediated inflammatory circumstance. Blocking NO production in MSCs increased the survival of intracellular mycobacteria, indicating NO-mediated antimycobacterial activity. Moreover, both nuclear factor κB (NF-κB) and Janus kinase (JAK)-signal transducer and activator of transcription (STAT) pathways were involved in iNOS expression and NO production in inflammatory microenvironment. Furthermore, pro-inflammatory cytokine interleukin-1β could trigger NO production in MSCs and exert anti-mycobacterial activity via NF-κB signaling pathway. Neutralization of interleukin-1β in macrophage-mediated inflammatory microenvironment dampened the ability of mouse MSCs to produce NO. Together, our findings demonstrated that macrophage-mediated inflammatory response during mycobacterial infection promotes the clearance of bacilli in mouse MSCs by increasing NO production, which may provide a better understanding of latent MTB infection.

*Mycobacterium tuberculosis* (MTB) infects approximately one-third of the global population and causes tuberculosis (TB), which is the leading bacterial cause of death worldwide^1^. MTB is an intracellular pathogen that can invade and survive within host macrophages^2^. Macrophages express several germ-line encoded pattern-recognition receptors such as Toll-like receptors (TLRs), which recognize conserved pathogen-associated molecular patterns (PAMPs) of MTB. Upon detection of invading MTB, macrophages initiate innate immune response and produce pro-inflammatory cytokines, such as interleukin-1β (IL-1β). In addition, activation of TLRs triggers direct antimicrobial mechanisms^3^, such as induction of autophagy^4^, production of antimicrobial peptide^5^ and generation of nitric oxide (NO)^6^,^7^,^8^, all of which play critical roles in the clearance of mycobacteria.

In mouse models of TB infection, the antimicrobial role of NO in host defense is well characterized^9^,^10^. Lipoproteins from MTB induce TLR-dependent NO production, which kills mycobacteria in macrophages *in vitro*^6^,^7^. NO production in macrophages requires inducible nitric oxide synthase (iNOS) expression, which could be regulated by PAMPs, pro-inflammatory cytokines (e.g. IL-1β) and interferons (IFNs)^11^. PAMPs and IL-1β trigger iNOS expression through nuclear factor κB (NF-κB) pathway^12^, while IFNs through Janus kinase (JAK)-signal transducer and activator of transcription (STAT) pathway^13^. iNOS catalyzes the conversion of amino acid L-arginine into NO in macrophages. The NO generated can react with essential structural components, key metabolic enzymes, nucleic acid or virulence factors of invading pathogens, and thus exert a strong antimicrobial effect^14^,^15^.

Although host cells employ multiple strategies to kill intracellular MTB, the eradication of this disease remains difficult. This has been largely attributed to the ability of MTB to maintain a latent or dormant infection in a host^16^. Latent MTB infection may persist for a lifetime of infected individuals, and reactivates to cause active disease in immunocompromised ones^17^. Therefore, it is both imperative and important to unravel the protective intracellular niches that enable MTB to survive in a latent state for a better understanding of the pathogenesis of TB and development of new drugs and vaccines.

During the asymptomatic infection phase, latent MTB may reside in a intracellular niche to maintain its viability^18^. Macrophages have been known for decades to serve as host cells for MTB^19^. However, *in vitro* study reveal that the viability of MTB in macrophages is low^20^; and no evidence exists indicating that these cells can maintain live MTB for a long time. Recent studies show that bone marrow mesenchymal stem cells (MSCs) may provide a protective niche for latent MTB. MTB could infect and survive in human and mouse MSCs *in vitro*^21^. After aerosol exposure, MTB disseminates rapidly from primary infection organ to mouse bone marrow, where it infects and resides in MSCs^22^. A well-characterized mouse model of TB dormancy shows that MTB may maintain long-term intracellular viability in MSCs *in vivo*^21^. Moreover, viable MTB is detected in MSCs isolated from individuals who have successfully completed months of anti-MTB chemotherapy, indicating that MSCs protect MTB from antimycobacterial drugs^23^. These findings demonstrate that MSCs may participate in the pathogenesis of latent TB and be responsible for TB reactivation. However, how inflammatory microenvironment during MTB infection regulate mycobacteria residency in MSCs is largely unknown.

The present study investigated the role of inflammatory microenvironment in regulating mycobacterial survival in mouse MSCs. Macrophage-mediated inflammatory microenvironment enhanced the clearance of mycobacteria residing in mouse MSCs. Under inflammatory condition, both NF-κB and JAK-STAT1 pathways were activated to induce NO production, which played a critical role in antimycobacterial activity in MSCs. Furthermore, we identified that IL-1β in inflammatory microenvironment was involved in the induction of NO and anti-mycobacterial activity in mouse MSCs. These findings may provide a better understanding of MTB infection in MSCs.

## Results

### Macrophage-conditioned inflammatory media decreased mycobacterial survival in mouse MSCs

**Figure 1:**
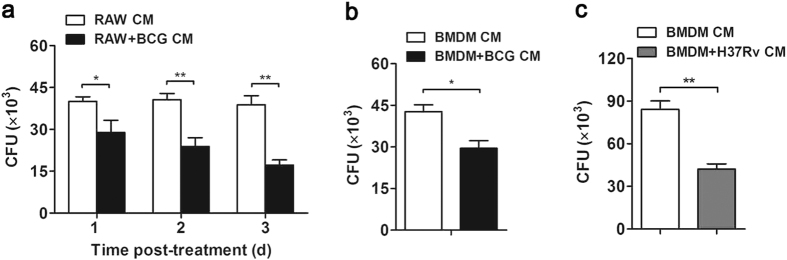
Macrophage-conditioned inflammatory media decreased survival of mycobacteria in mouse MSCs. MSCs were infected with *M. bovis* BCG or *M. tuberculosis* H37Rv (MOI 5) for 8 hr, followed by amikacin (200 μg/ml) treatment to remove extracellular bacteria. Mycobacteria-infected MSCs were treated with conditioned media from BCG-challenged RAW264.7 cells (RAW+BCG CM) (**a**), BCG-challenged BMDM (BMDM+BCG CM) (**b**) or H37Rv-challenged BMDM (BMDM+H37Rv CM) (**c**). The survival of mycobacteria in MSCs was determined by CFU assay. Data are shown as mean ± s.e.m. of three independent experiments. *p < 0.05; **p < 0.01.

To investigate the effect of macrophage-mediated inflammatory response on mycobacterial survival in MSCs, macrophage-like RAW264.7 cells were challenged with *M. bovis* BCG to elicit inflammatory response, and the conditioned media (CM) were collected to stimulate mouse MSCs harboring BCG. The survival of intracellular mycobacteria in MSCs was examined with colony-forming unit (CFU) assay. It was found that BCG-challenged macrophage-conditioned media (RAW+BCG CM) treatment decreased mycobacterial survival in MSCs at 1–3 days post-treatment as compared with uninfected macrophage-conditioned media (RAW CM) (Fig. 1a). Viable BCG in MSCs was decreased by 50% at 3 days post-treatment (Fig. 1a). Furthermore, conditioned media from BCG-challenged primary BMDM (BMDM+BCG CM) were collected to treat MSCs. Similarly, BMDM+BCG CM reduced the survival of BCG in MSCs to 70% as compared with conditioned media from uninfected BMDM (Fig. 1b). Moreover, we confirmed the observation above using virulent strain *M. tuberculosis* H37Rv. Consistently, conditioned media from H37Rv-challenged BMDM decreased intracellular H37Rv survival in MSCs by 50% (Fig. 1c). These results suggested that macrophage-mediated inflammatory media facilitated the clearance of mycobacteria in MSCs.

### Mouse MSCs produced elevated NO in macrophage-mediated inflammatory microenvironment

**Figure 2:**
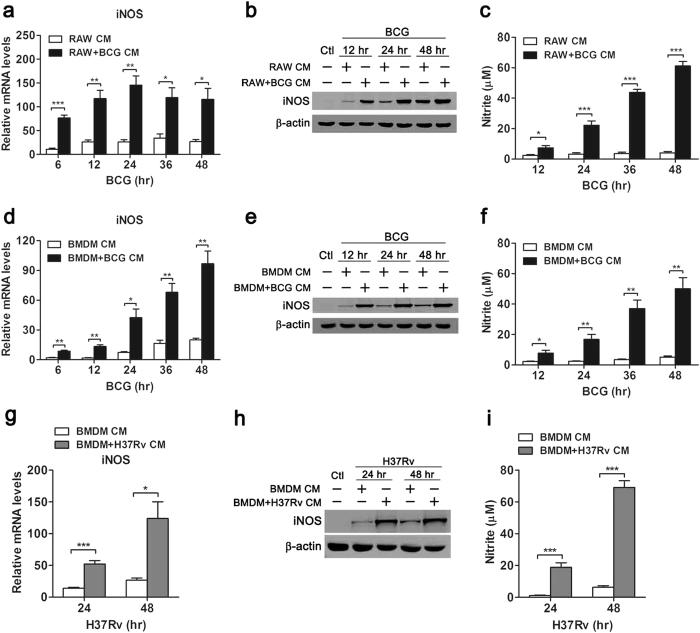
Increased iNOS expression and NO production of mouse MSCs in macrophage-mediated inflammatory microenvironment. MSCs were treated with conditioned media from BCG-challenged RAW264.7 cells (RAW+BCG CM) (**a**–**c**), BCG-challenged BMDM (BMDM+BCG CM) (**d**–**f**) or H37Rv-challenged BMDM (BMDM+H37Rv CM) (**g**–**i**), followed by BCG or H37Rv challenge. iNOS mRNA (**a**,**d**,**g**), protein expressions (**b**,**e**,**h**) and NO productions (**c**,**f**,**i**) were detected with real-time PCR, Western blot and Griess assay, respectively. Real-time PCR and Griess assay data are shown as mean ± s.e.m. of at least three independent experiments. Western blot data were representative of at least three experiments with similar results. *p < 0.05; **p < 0.01; ***p < 0.001.

To determine which antimicrobial mechanisms were induced in MSCs in inflammatory microenvironment, we examined the expression of iNOS and production of NO, which are well-characterized anti-mycobacterial mechanisms in mice. Real-time PCR data showed that RAW+BCG CM increased iNOS mRNA expression in MSCs by 2–4 folds as compared with untreated RAW264.7-conditioned media (RAW CM) (Fig. 2a). Similarly, the protein level of iNOS in RAW+BCG CM-treated MSCs was higher than that in RAW CM-treated MSCs as shown in the Western blot (Fig. 2b). Furthermore, NO production was detected by Griess assay. While very low levels of NO were produced in RAW CM-treated MSCs after BCG challenge, RAW+BCG CM dramatically induced NO production in MSCs (Fig. 2c). Consistently, BMDM+BCG CM significantly upregulated iNOS expression at both mRNA (Fig. 2d) and protein levels (Fig. 2e), and increased NO production (Fig. 2f) in MSCs. Furthermore, we examined iNOS expression and NO production using virulent MTB model. Similarly, H37Rv-triggered inflammatory response in macrophages enhanced iNOS mRNA and protein expression (Fig. 2g,h) as well as NO production in mouse MSCs (Fig. 2i). In addition, NO production was also determined in human cells, however, we failed to detect NO generation in human MSCs (data not shown), which was in accordance with previous reports^24^,^25^.

### NO mediated mycobactericidal activity in mouse MSCs under inflammatory condition

**Figure 3:**
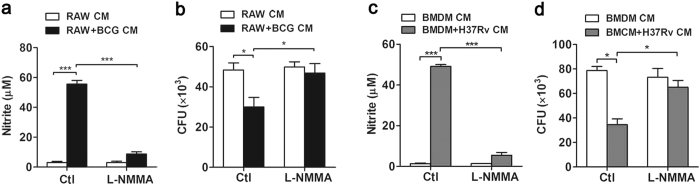
NO mediated mycobactericidal activity in mouse MSCs under inflammatory condition. BCG or H37Rv-infected MSCs were treated with conditioned media from BCG-challenged RAW264.7 cells (RAW+BCG CM) or H37Rv-challenged BMDM (BMDM+H37Rv CM) for 48 hr in the absence or presence of iNOS inhibitor (L-NMMA, 1 μM). NO production was detected with Griess assay (**a**,**c**). The survival of mycobacteria in MSCs was detected with CFU assay (**b**,**d**). Data are shown as mean ± s.e.m. of at least two independent experiments. *p < 0.05; ***p < 0.001.

To examine whether macrophage-mediated inflammatory media decreased mycobacterial survival in mouse MSCs via NO, iNOS inhibitor L-NMMA was used to block NO production. MSCs were pretreated with L-NMMA for 1 hr, followed by macrophage-conditioned inflammatory media stimulation. In RAW+BCG CM-treated MSCs, NO production was inhibited after L-NMMA treatment (Fig. 3a), confirming the efficacy of iNOS inhibition. We further tested the survival of BCG in MSCs after L-NMMA treatment using CFU assay. While RAW+BCG CM decreased bacterial load of BCG in MSCs, L-NMMA treatment attenuated the mycobactericidal activity (Fig. 3b). Moreover, virulent H37Rv strain was used to validate the involvement of NO in the clearance of mycobacteria in MSCs. Pharmacological inhibition of iNOS activity reduced NO production after BMDM+H37Rv CM treatment (Fig. 3c), and abolished the anti-mycobacterial effect of macrophage-mediated inflammatory media (Fig. 3d). These results indicated a critical role of NO in the containment of mycobacteria in mouse MSCs.

### NF-κB and JAK-STAT1 pathways were involved in NO production in mouse MSCs

**Figure 4:**
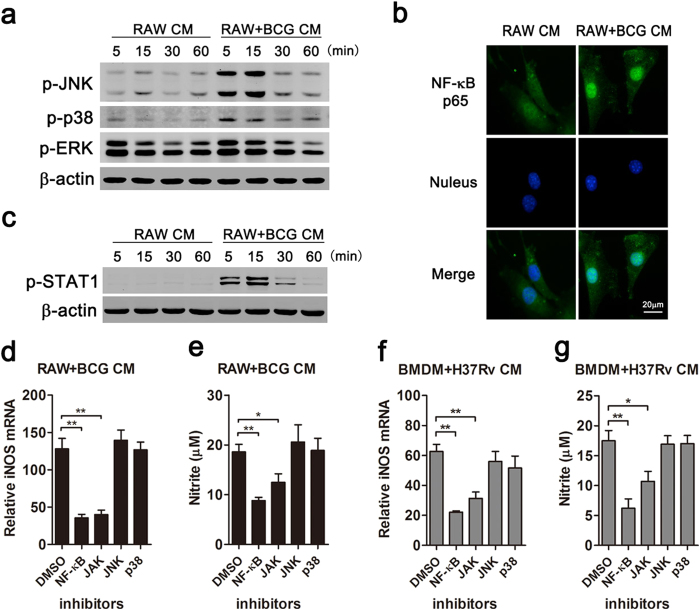
NF-κB and JAK-STAT1 pathways mediated NO production in mouse MSCs. MSCs were treated with conditioned media from BCG-challenged RAW264.7 cells (RAW+BCG CM) for the indicated time (**a**–**c**). The phosphorylation of JNK, p38, ERK (**a**) and STAT1 (**c**) was detected with Western blot. The nuclear translocation of NF-κB p65 was detected with immunofluorescence microscopy (**b**). MSCs were pre-treated with indicated inhibitors for 1 hr, followed by RAW+BCG CM or BMDM+H37Rv CM (**d**–**g**). The mRNA expressions of iNOS were detected with real-time PCR (**d**,**f**), and NO productions were measured with Griess assay (**e**,**g**). Data are shown as mean ± s.e.m. of at least three independent experiments. *p < 0.05; **p < 0.01.

To explore which pathways were responsible for NO production in MSCs, MAPK, NF-κB and JAK-STAT1 signalings were examined in MSCs under inflammatory condition. Western blot data revealed that RAW+BCG CM treatment increased the phosphorylation of JNK and p38 MAPK as compared with RAW CM, while the phosphorylation of ERK of the two groups was comparable (Fig. 4a). The activation of NF-κB pathway was detected by monitoring nuclear translocation of NF-κB p65 subunit with immunofluorescence microscopy. In RAW CM-treated MSCs, NF-κB p65 subunit was mainly distributed in the cytoplasm. However, NF-κB p65 protein aggregated in the nuclei of MSCs after RAW+BCG CM treatment (Fig. 4b). Moreover, RAW+BCG CM induced the phosphorylation of STAT1 in MSCs in a time-dependent manner (Fig. 4c). To determine which signalings mediated NO production, we further blocked each pathway with small molecule inhibitors. Real-time PCR data showed that inhibition of either NF-κB or JAK reduced iNOS induction after RAW+BCG CM treatment (Fig. 4c). Consistently, NO production was decreased in NF-κB or JAK inhibitor-treated MSCs (Fig. 4d). Nevertheless, blocking either JNK or p38 MAPK pathway did not alter iNOS expression (Fig. 4c) or NO production (Fig. 4d) in MSCs. In accordance, H37Rv-triggered inflammatory response in BMDM induced less iNOS expression (Fig. 4f) and NO production (Fig. 4g) in MSCs in the presence of NF-κB or JAK inhibitor. Collectively, these data suggested involvement of NF-κB and JAK-STAT1 pathways in NO production in mouse MSCs.

### IL-1β in macrophage-mediated inflammatory media induced NO production in mouse MSCs

**Figure 5:**
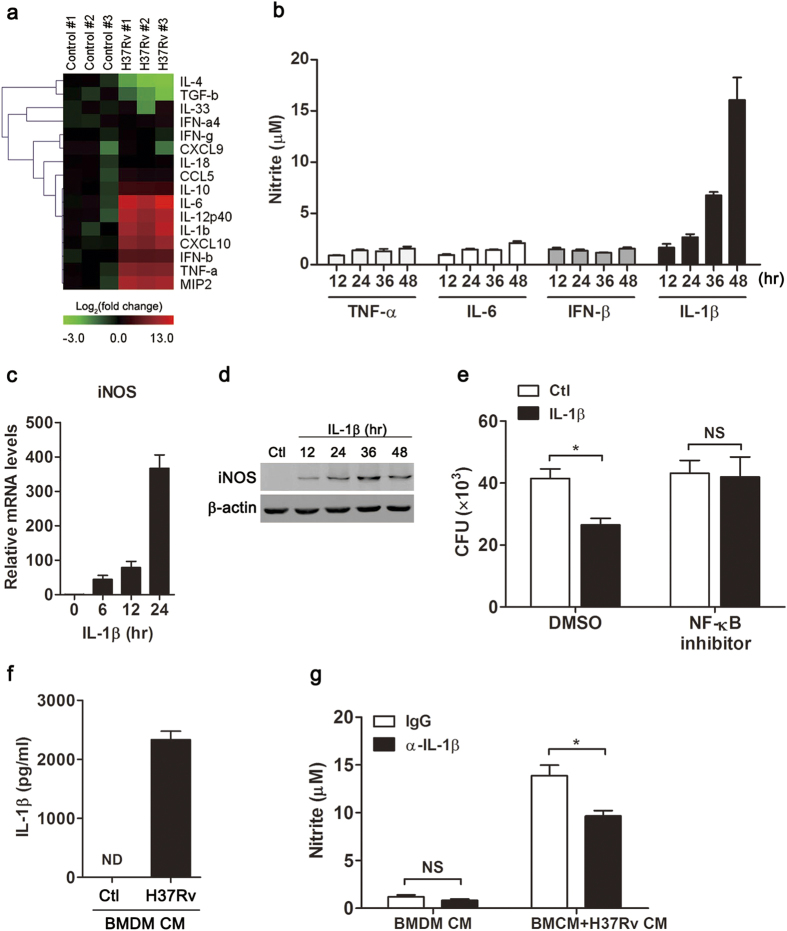
IL-1β in macrophage-mediated inflammatory media induced NO production in mouse MSCs. (**a**) Hierarchical cluster analysis of secreted factors differentially expressed between control and H37Rv-challenged BMDM. (**b**) MSCs were stimulated with indicated recombinant cytokines for 24 hr and NO productions were detected with Griess assay. iNOS mRNA (**c**) and protein (**d**) expressions were detected in IL-1β-treated MSCs. (**e**) The survival of mycobacteria in IL-1β-treated MSCs in the absence of presence of NF-κB inhibitor (10 μM) was detected with CFU assay. (**f**) The protein level of IL-1β in conditioned media from H37Rv-challenged BMDM was measured with ELISA. ND, not detectable. (**g**) Conditioned media from H37Rv-challenged BMDM were treated with IL-1β neutralizing antibody (α-IL-1β) or isotype IgG (40 μg/ml), and then used to treat MSCs. NO production was detected with Griess assay. Data are representative of at least two independent experiments. *p < 0.05; NS, not significant.

To determine which mediators in inflammatory microenvironment induced NO production in MSCs, we focused on genes coding for secreted molecules and performed PCR array analyses on control and H37Rv-challenged BMDM to identify differentially expressed genes (Fig. 5a). Among the genes upregulated upon H37Rv challenge, of interest were IL-1β, TNF-α and IFN-β, which are known to activate NF-κB and JAK-STAT1 pathways. Recombinant cytokines were used to treat MSCs, and productions of NO were determined by Griess assay. Only IL-1β among cytokines tested induced NO generation in MSCs (Fig. 5b). Both real-time PCR and Western blot analyses confirmed a time-dependent induction of iNOS in MSCs after IL-1β treatment (Fig. 5c,d). To establish the link between IL-1β and mycobacterial clearance in MSCs, we treated MSCs with exogenous IL-1β, and determined the survival of mycobacteria by CFU assay. A decrease in viable mycobacteria in MSCs was observed after IL-1β treatment, which was abolished in NF-κB inhibitor-treated cells (Fig. 5e). Moreover, H37Rv infection triggered IL-1β secretion in BMDM (Fig. 5f). To test the hypothesis that IL-1β in macrophage-mediated inflammatory media induced NO production, we blocked IL-1β with neutralizing antibody. Blocking IL-1β reduced NO production in BMDM+H37Rv CM-treated MSCs (Fig. 5g). Collectively, these results indicated that IL-1β in inflammatory microenvironment was involved in NO induction in MSCs during mycobacterial infection.

## Discussion

Previous studies reveal that MSCs serve as a protective niche for latent mycobacteria, which may be the underlying mechanism for TB reactivation^12^,^26^,^27^. However, the host defense against intracellular mycobacteria in MSCs is still unclear. Results of our study showed for the first time that macrophage-induced inflammatory microenvironment during mycobacterial infection promotes the clearance of mycobacteria in mouse MSCs through NO production.

Host cells employ several innate defense mechanisms, including autophagy, antimicrobial peptides and antimicrobial free radicals such as NO to eliminate intracellular mycobacteria. Autophagy is induced by several immunological factors^28^,^29^ and mediates innate immune responses against intracellular mycobacteria in macrophages by promoting maturation of mycobacterial phagosomes^4^,^30^. However, we found here that inflammatory microenvironment did not affect autophagy in MSCs (data not shown). In addition to autophagy, iNOS-derived NO is a well-defined effector in mouse macrophages that kills mycobacteria directly^15^,^31^. Lipoproteins of MTB stimulate transcription of iNOS and production of NO in a TLR-dependent manner, which leads to killing of intracellular MTB^6^,^7^. Mice deficient in iNOS gene show high susceptibility to MTB infection and exhibit disseminated disease^10^. In this study, we found that macrophage-induced inflammatory microenvironment triggered NO production in MSCs, which exerted antimycobacterial activity. Several studies have established that NO is a key mediator of immunosuppressive activity of MSCs^29^,^30^,^32^,^33^. Here, we demonstrated for the first time the antimicrobial function of NO in MSCs.

Several lines of evidence demonstrate that mouse and human macrophages have different anti-mycobacterial mechanisms. Activation of TLR with mycobacterial lipoprotein mediated an NO-independent antimicrobial activity in mouse primary macrophages and cell lines^6^,^7^,^10^,^34^. However, in primary human monocytes and macrophages, TLR-induced antimicrobial activity was not dependent on iNOS activity, and NO could not be detected in these cells^6^. Following studies revealed that TLR2 activation of human macrophages up-regulated expression of the vitamin D receptor and the vitamin D-1–hydroxylase genes, which mediated induction of the antimicrobial peptide cathelicidin to kill intracellular MTB^5^,^35^. Similar to macrophages, MSCs show differential immunomodulatory mechanisms between mouse and human. While high level of NO is produced in mouse MSC after activation with IL-1β and IFN-γ^25^,^32^,^33^, human MSCs do not express iNOS or produce NO^24^,^36^. For human cells model, we found that human MSCs did not produce NO after inflammatory conditioned media treatment (data not shown). Therefore, we have restricted our conclusion to mouse cell model in this report. Whether human microphages modulate anti-mycobacterial activity in human MSCs via other mechanism needs further investigation.

Generation NO requires iNOS expression which converts L-arginine and oxygen into L-citrulline and NO. iNOS induction is regulated by several signaling pathways and transcription factors. Activation of the transcription factors NF-κB and STAT1 and subsequent binding to the iNOS promoter are essential steps for iNOS expression in most cells^12^. It has been well characterized that NF-κB pathway is critical to the induction of iNOS in macrophages by PAMPs, such as LPS^37^. However, studies have revealed that full induction of iNOS expression and NO production requires activation of JAK-STAT pathway^13^. A recent study demonstrated that NF-κB and STAT cooperate to promote recruitment of RNA polymerase II to the iNOS transcription start site to initiate gene transcription. This study found that blocking either NF-κB or JAK-STAT pathway reduced iNOS expression and NO production in mouse MSCs in response to inflammatory microenvironment. Previous studies showed that MAPK pathways were involved in the induction of iNOS in macrophages. Bhatt *et al.* reported that JNK positively regulated peptidoglycan-induced iNOS expression and NO production, while ERK served as a negative regulator^38^. Inhibition of p38 MAPK significantly impaired iNOS expression in RAW264.7 cells after LPS stimulation^39^. In this study, macrophage-conditioned inflammatory media promoted JNK and p38 MAPK activation in MSCs. However, blocking neither JNK nor p38 MAPK affected NO production in mouse MSCs in response to macrophage-conditioned inflammatory media. These findings suggest a context-dependent role of MAPK in induction of iNOS.

Several pro-inflammatory cytokines, IFNs and microbial products are potent inducer of iNOS expression in macrophages. Pro-inflammatory cytokine IL-1β signals through IL-1 receptor (IL-1R) and MyD88, which activates NF-κB pathway to trigger iNOS-mediated production of NO^31^. Inflammasome-driven IL-1β production facilitated host resistance to Leishmania infection through NO production^40^. IFNs elicit the phosphorylation and the dimerization of STAT1, while engagement of PRRs with microbial ligands leads to the activation and the nuclear translocation of NF-κB^12^. Therefore, IFNs and bacterial components act cooperatively to trigger iNOS expression. Previous study showed that iNOS expression was induced in MSCs after recombinant IL-1β and IFN-γ treatment, and neutralization of IL-1β or IFN-γ reduced iNOS expression and NO production in MSCs co-cultured with activated T cells^33^. Here, we detected IL-1β secretion in H37Rv-challenged macrophages, and found that blocking IL-1β reduced NO production in MSCs. Although the phosphorylation of STAT1 was observed in MSCs treated with macrophage-mediated inflammatory media, we failed to detect IFN-γ production in the media by ELISA (data not shown). Increased expression of IFN-β was detected in H37Rv-challenged macrophages, which can also activate transcription factor STAT1. However, recombinant IFN-β did not induced NO production in MSCs. Therefore, we speculate that other cytokines triggering JAK-STAT signaling induce iNOS expression in synergy with NF-κB signaling, which needs further investigation to identify.

In addition to multipotentiality, MSCs possess immunosuppressive properties. MSCs promote differentiation of monocytes towards anti-inflammatory macrophages and induce macrophage M2 polarization^27^,^41^. Moreover, MSCs enhance the generation of regulatory T cells, which inhibit inflammatory response indirectly^26^. Therefore, it is conceivable that MSCs may inhibit macrophage-mediated inflammatory response in mycobacterial infection site *in vivo*, which hinders elimination of mycobacteria harboring in MSCs and in turn facilitates bacterial evasion of host immunity.

In summary, results of our study showed that macrophage-mediated inflammatory microenvironment during mycobacterial infection facilitated the clearance of intracellular H37Rv and BCG in mouse MSCs via NO. Under inflammatory circumstance, NK-κB and JAK-STAT1 pathways were activated in mouse MSCs, which ultimately resulted in NO production. Furthermore, we revealed that pro-inflammatory cytokine IL-1β was involved in the production of NO and anti-mycobacterial activity through NF-κB signaling in mouse MSCs. These findings provided a better understanding of the pathogenesis of latent infection of MTB.

## Methods

### Ethics statement

All experimental protocols and methods were approved by Sun Yat-sen University, and were carried out in accordance with the approved guidelines. All animal experiments were approved by the Animal Ethics Committee of Sun Yat-sen University and performed in accordance with the guidelines of Animal Care and Use of Sun Yat-sen University.

### Reagents

Anti-phospho-JNK, anti-phospho-ERK and anti-phospho-p38 MAPK antibodies were obtained from Cell Signaling Technology (Beverly, MA). Anti-JNK, anti-ERK and anti-p38 MAPK antibodies were from Santa Cruz Biotechnology (Santa Cruz, CA). Anti-iNOS antibody was from eBioscience (San Diego, CA). Anti-β-actin antibody was from Sigma (St. Louis, MO). NF-κB inhibitor (JSH-23), JNK inhibitor (SP600125) and p38 MAPK inhibitor (SB 203580) were from Merck (Darmstadt, Germany). JAK inhibitor (Ruxolitinib) was obtained from Selleck Chemicals (Houston, TX). iNOS inhibitor (L-NMMA) was from Sigma (St. Louis, MO). Recombinant mouse IL-1β was obtained from PeproTech (Rocky Hill, NJ). Mouse IL-1β neutralizing antibody was from R&D Systems (Minneapolis, MN). Middlebrook 7H9 broth medium and Middlebrook 7H10 agar were purchased from BD Difco Laboratories (Sparks, MD).

### Cell culture

Mouse MSCs were isolated from bone marrow of C57BL/6 mice and immunophenotyped by flow cytometry as described previously^42^,^43^,^44^. Briefly, MSCs are adherent to plastic culture flask with fibroblast-like shape, and could differentiate into adipocytes, osteoblasts and chondroncytes in specified culture media. For surface markers, mouse MSCs express Sca-1, CD105, CD73 and CD90, but are negative for CD34, CD45 and CD11b^45^. Cells were maintained in DMEM low glucose medium supplemented with 10% FBS, 2 mM glutamine, 100 U/ml penicillin and 100 mg/ml streptomycin (all from Invitrogen, Carlsbad, CA). MSCs from passage 5 to 20 were used in all experiments. Macrophage-like RAW264.7 cells were maintained in DMEM supplemented with 10% FBS as reported previously^46^. Bone marrow-derived macrophages (BMDMs) were isolated and cultured as described previously^47^. All experiments involving animals were approved by the Animal Ethics Committee of Sun Yat-sen University.

### Bacterial culture

*M. bovis* BCG strain 19015 and *M. tuberculosis* H37Rv strain 25618 were purchased from the American Type Culture Collection (ATCC), and was grown in Middlebrook 7H9 broth medium or on 7H10 agar plates supplemented with 10% OADC at 37 °C. *M. bovis* BCG or *M. tuberculosis* H37Rv was homogenized to generate a single cell suspension to infect cells as reported previously^48^. Experiments using virulent strain (H37Rv) were carried out in the Center for Tuberculosis Control of Guangdong Province (Guangzhou, China).

### Conditioned media preparation

RAW264.7 cells or BMDMs were seeded in 12-well plates (2.5 × 10^5^ cells/well) one day before infection. On the second day, cells were refed with fresh culture medium (DMEM supplemented with 10% FBS, 1 ml/well) and then infected with *M. bovis* BCG or *M. tuberculosis* H37Rv at a multiplicity of infection (MOI) of 5. At 24 hr post-infection supernatants were collected, centrifuged to remove cell debris and sterilized with 0.22 μm filter to prepare conditioned media. Aliquots of conditioned media were stored in −80 °C freezer before use within one week.

### Detection of mycobacteria survival in MSCs

MSCs were infected with *M. bovis* BCG or *M. tuberculosis* H37Rv at a multiplicity of infection (MOI) of 5 for 8 hr. The infected cells were treated with amikacin (200 μg/ml) for 3 hr to remove extracellular bacteria without disturbing the growth of intracellular bacilli as described previously^49^. The infected MSCs were treated with conditioned media for the indicated time, and then the cells were lysed with sterile water containing 0.01% Triton X-100. Serial 10-fold dilution was prepared, and the diluted cell lysate was plated on Middlebrook 7H10 agar plates. The agar plates were incubated for 3 weeks at 37 °C with 5% CO_2_, and colony forming units (CFU) were counted.

### Griess assay

Nitric oxide production was determined by measuring its stable end product nitrite, using a Griess reagent (Promega Corporation, Madison, WI) according to manufacturer’s protocol. Briefly, 50 μl of supernatant was added to 96-well plate, followed by 50 μl sulphanilamide and 50 μl N-1-napthylethylenediamine dihydrochloride (NED). Absorbance at 540 nm was measured by microplate reader and nitrite concentrations were estimated using a standard nitrite curve. For nitric oxide production in conditioned-media-treated MSCs, nitrite in corresponding conditioned media was subtracted from that in MSCs supernatant.

### Real-time RT-PCR and PCR array data analysis

Total RNA was extracted from cells with TRIzol Reagent (Life Technologies, Carlsbad, CA) and reverse transcribed into cDNA by RevertAid™ First Strand cDNA Synthesis Kit (Thermo Fisher Scientific, Waltham, MA) as described previously^50^,^51^,^52^. Quantitative real-time PCR analysis of transcripts was performed on Bio-Rad CFX96 real-time detection system using SYBR Green Master Mix (Applied Biosystems, Foster City, CA)^53^,^54^. Relative mRNA expression levels were calculated after normalization to β-actin. Sequences of primer pairs used are shown in Table 1 or described previously^42^,^43^,^47^. For PCR array analysis, relative gene expression levels were calculated compared with the average value of control group, and log2 of fold change was shown. Hierarchical clustering was performed with the MultiExperiment Viewer (MeV) program (version 4.9.0) using Pearson correlation and average linkage.

### Western blot

Western blot was performed as described previously^55^,^56^. Briefly, the whole-cell extract was resolved by SDS-polyacrylamide gel electrophoresis and transferred to nitrocellulose membranes. The membranes were blocked in 5% bovine serum albumin and then incubated with diluted primary antibodies at 4 °C overnight. Western blot detection was performed with IRDye 800 CW conjugated anti-rabbit IgG or IRDye 680 CW conjugated anti-mouse IgG secondary antibodies according to the manufacturer’s protocols (LI-COR Biosciences, Lincoln, NE). The blots were visualized using Odyssey infrared imaging system (LI-COR Biosciences).

### Immunofluorescence microscopy

Immunofluorescence microscopy was performed as described previously^57^. Briefly, cells were grown on glass coverslips and treated as indicated, and then fixed, permeabilized and blocked. Samples were incubated with primary antibody at 4 °C overnight, and then with secondary antibody for 1 hr at room temperature. Nuclei were labeled with 4,6-diamidino-2-phenylindole (DAPI) staining. Coverslips were mounted with ProLong Gold antifade reagent (Invitrogen) and visualized using Olympus BX53 fluorescence microscope (Olympus Corporation, Tokyo, Japan).

### Enzyme-linked immunosorbent assay (ELISA)

Supernatants from H37Rv-challenged and control BMDM were harvested, and IL-1β protein levels were tested using ELISA kit (BD OptEIA^TM^ Mouse IL-1β ELISA Set, BD Bioscience) following the manufacturer’s instructions. The detection limit of IL-1β is 15.6 pg/ml.

### Statistical analysis

Data are shown as mean ± s.e.m. Statistical analysis was performed using GraphPad Prism 5.0 (GraphPad Software, San Diego, CA). Differences between two groups were compared by using Student’s *t*-test. Differences with a *p* value less than 0.05 were considered statistically significant.

## Additional Information

**How to cite this article**: Yang, K. *et al.* Macrophage-mediated inflammatory response decreases mycobacterial survival in mouse MSCs by augmenting NO production. *Sci. Rep.* **6**, 27326; doi: 10.1038/srep27326 (2016).

## Figures and Tables

**Table 1:** Sequence of primers used in PCR.

Gene	Primer Sequence (5′-3′)
IL-1β	Fwd: CGCAGCAGCACATCAACAAGAGC
	Rev: TGTCCTCATCCTGGAAGGTCCACG
IL-4	Fwd: GCAACGAAGAACACCACAGAGAGT
	Rev: GGCATCGAAAAGCCCGAAAGAGT
IL-10	Fwd: AGCTGGACAACATACTGCTAACCGAC
	Rev: CTTGATTTCTGGGCCATGCTTCTCTG
IL-12 p40	Fwd: GGTCACACTGGACCAAAGGGACTATG
	Rev: ATTCTGCTGCCGTGCTTCCAAC
MIP-2	Fwd: TGTCAATGCCTGAAGACCCTGCC
	Rev: AACTTTTTGACCGCCCTTGAGAGTGG
IFN-γ	Fwd: GTTACTGCCACGGCACAGTCATTG
	Rev: ACCATCCTTTTGCCAGTTCCTCCAG
IFN-α4	Fwd: GAGAAGGTGGATAACCAACAG
	Rev: GGAGGTCATTGCAGAATGAG
TGF-β1	Fwd: TACGTCAGACATTCGGGAAGCA
	Rev: CCAAGGTAACGCCAGGAATTGT
CCL5	Fwd: GAACCGCCAAGTGTGTGCCA
	Rev: GGCTAGGACTAGAGCAAGCAATGA
CXCL9	Fwd: GCATCATCTTCCTGGAGCAGTGT
	Rev: TGAGGGATTTGTAGTGGATCGT
CXCL10	Fwd: CTTCTGAAAGGTGACCAGCCGT
	Rev: GTCGCACCTCCACATAGCTTAC
